# Supplementary material for: Implementation and evaluation of a dynamic contrast-enhanced MR perfusion protocol for glioblastoma using a 0.35 T MRI-Linac system
Source: Phys Med. Author manuscript; Available in PMC 2025 Mar 1. (PMC11575850; doi:10.1016/j.ejmp.2024.103316)
Supplement: Supplementary material [file NIHMS2021631-supplement-Supplementary_material.docx]

| Table S1 - Patient 1 | | | | | | |
| --- | --- | --- | --- | --- | --- | --- |
|  | Pre RT | | Mid RT | | Post RT | |
|  | VR | 3T | VR | 3T | VR | 3T |
| Structures | Volumes in cc | | | | | |
| '3rd Ventricle' | 0.92 | 0.96 | 1.46 | 1.72 | 2.05 | 1.93 |
| 'Right Accumbens Area' | 0.71 | 0.75 | 0.35 | 0.51 | 0.38 | 0.43 |
| 'Left Accumbens Area' | 0.29 | 0.50 | 0.24 | 0.48 | 0.36 | 0.41 |
| 'Right Amygdala' | 0.83 | 1.15 | 0.83 | 1.17 | 0.78 | 0.92 |
| 'Left Amygdala' | 0.50 | 0.98 | 0.52 | 0.92 | 0.62 | 0.85 |
| 'Right Inf Lat Vent' | 0.00 | 0.07 | 0.27 | 0.24 | 0.36 | 0.33 |
| 'Left Inf Lat Vent' | 0.14 | 0.10 | 0.04 | 0.19 | 0.27 | 0.25 |
| 'Right Pallidum' | 0.65 | 0.78 | 0.31 | 0.32 | 0.26 | 0.33 |
| 'Left Pallidum' | 0.36 | 0.37 | 0.26 | 0.32 | 0.25 | 0.34 |
| 'Right Putamen' | 5.62 | 6.04 | 3.87 | 3.59 | 3.41 | 3.10 |
| 'Left Putamen' | 1.66 | 2.74 | 2.87 | 2.74 | 2.48 | 2.45 |
| 'Right Thalamus Proper' | 4.83 | 3.52 | 3.94 | 3.28 | 3.91 | 3.36 |
| 'Left Thalamus Proper' | 4.64 | 3.52 | 3.82 | 3.52 | 4.46 | 3.66 |
| 'Left Basal Forebrain' | 0.37 | 0.43 | 0.43 | 0.47 | 0.39 | 0.46 |
| 'Right Basal Forebrain' | 0.74 | 0.66 | 0.45 | 0.54 | 0.46 | 0.50 |
| 'Right ACgG anterior cingulate gyrus' | 7.26 | 5.64 | 4.53 | 4.80 | 4.31 | 3.80 |
| 'Left ACgG anterior cingulate gyrus' | 6.28 | 6.20 | 5.05 | 5.56 | 4.73 | 5.55 |
| 'Right FO frontal operculum' | 3.40 | 3.75 | 2.32 | 2.48 | 2.02 | 2.21 |
| 'Left FO frontal operculum' | 1.30 | 1.94 | 1.50 | 1.95 | 1.34 | 1.82 |
| 'Right LOrG lateral orbital gyrus' | 2.09 | 2.43 | 2.01 | 1.92 | 1.75 | 1.81 |
| 'Left LOrG lateral orbital gyrus' | 1.77 | 2.33 | 2.14 | 2.37 | 2.15 | 2.16 |
| 'Right MOrG medial orbital gyrus' | 4.61 | 5.44 | 4.59 | 4.38 | 4.41 | 4.55 |
| 'Left MOrG medial orbital gyrus' | 4.30 | 4.63 | 4.50 | 4.47 | 4.42 | 4.45 |
| 'Right OrIFG orbital part of the inferior frontal gyrus' | 2.14 | 2.23 | 1.32 | 1.52 | 1.19 | 1.27 |
| 'Left OrIFG orbital part of the inferior frontal gyrus' | 1.31 | 1.57 | 1.31 | 1.54 | 1.36 | 1.39 |
| 'Left PHG parahippocampal gyrus' | 2.49 | 2.86 | 1.13 | 2.70 | 2.76 | 2.69 |
| 'Right PIns posterior insula' | 3.96 | 3.28 | 2.33 | 2.42 | 2.06 | 2.15 |
| 'Left PIns posterior insula' | 1.35 | 2.15 | 2.09 | 2.12 | 1.99 | 2.06 |
| 'Right PP planum polare' | 2.17 | 2.62 | 1.93 | 2.32 | 1.83 | 2.01 |
| 'Left PP planum polare' | 1.77 | 2.40 | 2.12 | 2.34 | 2.00 | 2.31 |
| 'Right SCA subcallosal area' | 1.74 | 1.81 | 1.50 | 1.58 | 1.41 | 1.73 |
| 'Left SCA subcallosal area' | 1.78 | 1.83 | 1.52 | 1.62 | 1.44 | 1.61 |
| 'Right TTG transverse temporal gyrus' | 1.82 | 1.66 | 1.45 | 1.56 | 1.25 | 1.51 |
| 'Left TTG transverse temporal gyrus' | 1.30 | 1.83 | 1.45 | 1.88 | 1.50 | 1.69 |
|  |  |  |  |  |  |  |

| Table S2 - Patient 2 | | | | |
| --- | --- | --- | --- | --- |
|  | Pre RT | | Mid RT | |
|  | VR | 3T | VR | 3T |
| Structures | Volumes in cc | | | |
| '3rd Ventricle' | 0.89 | 0.69 | 0.84 | 0.93 |
| 'Right Accumbens Area' | 0.50 | 0.41 | 0.42 | 0.41 |
| 'Left Accumbens Area' | 0.56 | 0.42 | 0.47 | 0.46 |
| 'Right Caudate' | 3.55 | 2.80 | 4.45 | 3.98 |
| 'Left Caudate' | 3.08 | 2.63 | 2.50 | 2.95 |
| 'Right Pallidum' | 0.48 | 0.46 | 0.60 | 0.65 |
| 'Left Pallidum' | 0.55 | 0.39 | 0.58 | 0.60 |
| 'Right Putamen' | 2.69 | 2.33 | 2.23 | 2.13 |
| 'Left Putamen' | 2.78 | 2.54 | 2.42 | 2.12 |
| 'Right Thalamus Proper' | 2.85 | 2.95 | 3.17 | 3.21 |
| 'Left Thalamus Proper' | 3.82 | 3.26 | 3.83 | 3.40 |
| 'Right AIns anterior insula' | 4.04 | 3.87 | 3.69 | 4.03 |
| 'Left AIns anterior insula' | 3.92 | 3.96 | 3.79 | 4.05 |
| 'Right CO central operculum' | 3.90 | 3.64 | 3.08 | 3.94 |
| 'Left CO central operculum' | 3.80 | 3.94 | 3.69 | 4.09 |
| 'Right FO frontal operculum' | 1.92 | 1.89 | 1.50 | 1.88 |
| 'Left FO frontal operculum' | 1.63 | 1.77 | 1.46 | 1.79 |
| 'Right PCgG posterior cingulate gyrus' | 3.08 | 2.83 | 2.98 | 3.42 |
| 'Left PCgG posterior cingulate gyrus' | 3.68 | 3.39 | 3.84 | 3.92 |
| 'Right PIns posterior insula' | 2.47 | 2.15 | 2.03 | 2.12 |
| 'Left PIns posterior insula' | 2.02 | 2.02 | 1.96 | 1.97 |
| 'Right PP planum polare' | 1.53 | 1.49 | 1.59 | 1.83 |
| 'Left PP planum polare' | 1.62 | 1.72 | 1.73 | 1.93 |
| 'Right SMC supplementary motor cortex' | 5.00 | 4.15 | 5.48 | 5.67 |
| 'Left SMC supplementary motor cortex' | 4.97 | 4.54 | 4.88 | 5.97 |
| 'Right SOG superior occipital gyrus' | 2.58 | 2.95 | 2.53 | 3.27 |
| 'Left SOG superior occipital gyrus' | 2.03 | 2.66 | 2.18 | 2.74 |
| 'Right TTG transverse temporal gyrus' | 1.32 | 1.01 | 0.83 | 1.12 |
| 'Left TTG transverse temporal gyrus' | 1.31 | 1.35 | 1.27 | 1.25 |
